# Optimization of Polyplex Formation between DNA Oligonucleotide and Poly(l-Lysine): Experimental Study and Modeling Approach

**DOI:** 10.3390/ijms18061291

**Published:** 2017-06-17

**Authors:** Tudor Vasiliu, Corneliu Cojocaru, Alexandru Rotaru, Gabriela Pricope, Mariana Pinteala, Lilia Clima

**Affiliations:** 1 Center of Advanced Research in Bionanocojugates and biopolymers, “Petru Poni” Institute of Macromolecular Chemistry, Iasi, Romania Aleea Grigore Ghica Voda 41A, 70487 Iasi, Romania; vasiliu.tudor@icmpp.ro (T.V.); rotaru.alexandru@icmpp.ro (A.R.); pricope.gabriela@icmpp.ro (G.P.); pinteala@icmpp.ro (M.P.); 2 Department of Inorganic Polymers, “Petru Poni” Institute of Macromolecular Chemistry, Iasi, Romania Aleea Grigore Ghica Voda 41A, 70487 Iasi, Romania; cojocaru.cornliu@icmpp.ro

**Keywords:** DNA, modeling, optimization, poly(l-Lysine)

## Abstract

The polyplexes formed by nucleic acids and polycations have received a great attention owing to their potential application in gene therapy. In our study, we report experimental results and modeling outcomes regarding the optimization of polyplex formation between the double-stranded DNA (dsDNA) and poly(l-Lysine) (PLL). The quantification of the binding efficiency during polyplex formation was performed by processing of the images captured from the gel electrophoresis assays. The design of experiments (DoE) and response surface methodology (RSM) were employed to investigate the coupling effect of key factors (pH and N/P ratio) affecting the binding efficiency. According to the experimental observations and response surface analysis, the N/P ratio showed a major influence on binding efficiency compared to pH. Model-based optimization calculations along with the experimental confirmation runs unveiled the maximal binding efficiency (99.4%) achieved at pH 5.4 and N/P ratio 125. To support the experimental data and reveal insights of molecular mechanism responsible for the polyplex formation between dsDNA and PLL, molecular dynamics simulations were performed at pH 5.4 and 7.4.

## 1. Introduction

Gene therapy is a medical procedure that involves the insertion of nucleic acids into cells, thus altering the gene expression in order to correct gene defects [1]. There are mainly two approaches to gene therapy: one that uses viral vectors as means of transporting the genetic material [2,3,4] and one that uses cationic non-viral vectors [5,6]. In fact, ~70% of gene therapy clinical trials carried out so far have used modified viruses such as retroviruses, lentiviruses, adenoviruses and adeno-associated viruses (AAVs) to deliver genes; however the use of viruses for gene therapy has a set of disadvantages related to the inactivation and their modification [7]. Non-viral DNA delivery systems, on the other side, have attracted considerable attention in the last decade not only for fundamental research interests but also for applications in clinical trials [7]. The common advantages of non-viral vectors are the inferior specific immune responses, and they are generally safer and easier to design and synthesize, with more flexible structures and chemical properties for various purposes [8,9,10,11]. The main problem for the clinical use of non-viral vectors is their low transfection efficacy [12]. Often, the synthetic pathway of non-viral vectors needs to be adjusted and optimized in order to obtain the needed gene delivery properties. Cationic polymers are frequently used for the preparation of non-viral vectors due to their capacity to easily interact and bind nucleic acids. The most studied polymers for the preparation of non-viral vectors are linear or branched poly (ethylene imine) (PEI) and polypeptide-type poly(l-Lysine) (PLL) [11,13]. PEI, depending on its structure, constitutes a high concentration of positively charged amine groups (primary, secondary, tertiary), which enables effective electrostatic binding and condensation of negatively charged DNA [14] and possesses buffering capacity and polymer swelling at the acidic pH of the endosomes [10]. In the case of PLL, the polymer chemical structure consists of only primary amines in the side chains, which take part in binding DNA. Absence of the proton sponge ability of PLL, together with the aggregation and precipitation of the PLL-DNA complexes at high NaCl concentrations [15], considerably diminish gene transfection at the cellular level and makes PLL inferior candidate when compared to PEI. Even though the PLL expresses lower gene transfection, it still has excellent characteristics as a gene carrier and proved to be more advantageous in comparison to PEI in terms of cytotoxicity [16]. It is still unclear if the differences in transfection efficiency of polyplexes formed from nucleic acids and PEI or PLL are caused by dissimilarities in their affinities to double-stranded DNA (dsDNA), their chemical structure or polyplex structures. Revealing these structure–activity relationships is very important for controlling the functionality of novel biomaterials to be used for gene therapy.

Ziebarth et al. have performed theoretical molecular dynamics (MD) simulations of the short DNA duplex in the presence of PEI or PLL to shed light on the specific atomic interaction that results in the formation of polyplexes [17]. It was found that, in comparison with PLL, PEI is able to better neutralize the charge of dsDNA. In another study, an experimental step towards understanding the mechanisms of dsDNA complexation behavior of PEI and PLL was performed by Ketola et al. [18]. The authors investigated the PEI and dsDNA and PLL and dsDNA (dsDNA/PLL) complex formation at different pH values using a time-resolved spectroscopic method. It was observed that pH and N/P ratio (expressed by nitrogen/phosphorus ratio, where N represents the content of nitrogen in one polymeric unit of cationic polymer (PEI or PLL) and P represents content of phosphates within the DNA backbone) have a clear effect on the mechanism of polyplex formation for the studied polymers, these parameters determining the independent or cooperative types of the binding mechanisms. Since pH is an important factor in both binding and dissociation of the formed polyplexes, it is crucial to explore in depth the interaction of various cationic macromolecules with nucleic acids at the physiological pH range (5.0–7.4).

Recently, we have studied the optimization of polyplex formation between short dsDNA oligonucleotide and branched PEI at different pH values [19]. A design of experiments was adopted to investigate the binding efficiency of DNA and branched PEI under various conditions of components ratio and the pH of the solution. Additionally, the molecular dynamic simulation of the investigated complexation process at pH = 7.4, in order to unveil the mechanism of polycomplex formation at atomic-scale was performed.

In the present study, we continue to investigate the mechanisms of polyplex formation by optimizing the short dsDNA complexation by PLL at different physiological pH values and performing MD simulation of the complexation process at two different pH values (pH = 5.4 and 7.4). In recent years, several research groups have also addressed the MD simulations of dsDNA/PLL complexes using a Drew–Dickerson dodecamer d(CGCGAATTCGCG)_2_ as the model for the short DNA helix [20]. Besides the work of Ziebarth et al. [17], who investigated the molecular dynamics simulations of DNA duplex in the presence of PLL at pH = 7.4, no additional dynamic simulations of PLL with nucleic acids were performed in order to compare results at various pH values.

In this study, the optimization of the polyplex formation process between PLL and dsDNA was accomplished by keeping constant the DNA concentration and varying the amount of PLL and pH value. Experiments were performed utilizing gel electrophoresis data as read out results. We employed the design of experiments (DoE) and response surface methodology (RSM) for process modeling and optimization. These statistical tools have been widely accepted and applied for investigation, modeling and optimization of various biotechnological processes [21,22,23,24,25,26].

## 2. Results and Discussions

### 2.1. Data-Driven Modeling and Optimization of the Polyplex Formation Process

The polyplex formation has a set of optimum reaction conditions in which the efficiency is at a maximum value, and, in order to determine the exact values of these conditions, the response surface methodology (RSM) [27,28,29,30,31] can be used. In this study, the design of experiments (DoE) and RSM was used to quantitatively determine the complexation between dsDNA and PLL, using two input variables, i.e., pH of the solution and N/P ratio. To facilitate the modeling process, the input variables were converted to coded variables: −1 for the minimum level, +1 for the maximum level and 0 for the central level (Table 1).

**Figure 1:**
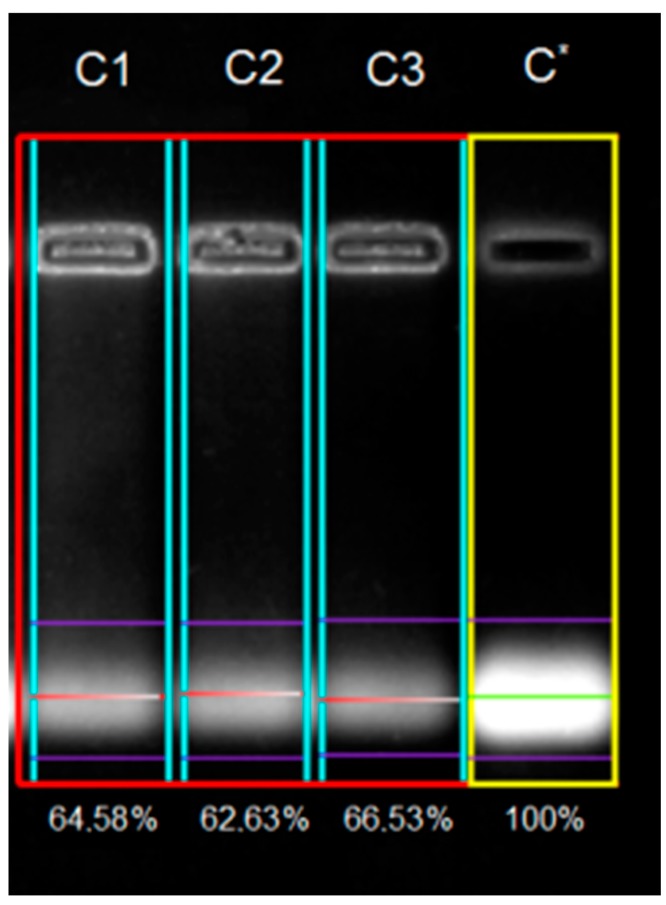
Example of gel electrophoresis run performed at central point (pH 6.4) using Gel Quant Express software (version, Manufacturer, City, US State abbrev. if applicable, Country). Lanes C1, C2 and C3 indicate loaded samples with N/P = 75; bright bands in the well (**top**) correspond to the formed polyplex, and the lower migrated bands (**bottom**) correspond to the unbound dsDNA. Lane C* represents a reference dsDNA sample with an associated signal intensity of 100%.

This conversion scheme was done to simplify the use of variables and to apply the same non-dimensional scale for all of the factors [27,28,29,30,31]. The actual values of these coded variables are summarized in Table 1. Likewise, the faced-centered experimental design used to study the complexation process is presented in Table 2. In proposed experiments, the concentration of dsDNA in the sample was kept constant (26.28 μM), and the desired N/P ratio was achieved by varying the amount of PLL in the sample. Polyplexes with N/P ratio of 25, 75 and 125 were prepared at three different pH values 5.4, 6.4 and 7.4. An agarose gel electrophoresis retardation assay was used to evaluate the binding between PLL and dsDNA sequence at different pH values and various N/P ratios (Figure 1 and Figures S1–S3). Table 2 summarizes 11 gel electrophoresis experimental runs, comprising factorial (F1–F4), axial (A1–A4) and central points (C1–C3) according to DoE terminology.

Note that, central points (C1–C3) were carried out to test the reproducibility of the method under the same experimental conditions. Figure 1 illustrates an example of the gel electrophoresis assay determined for the central points (C1–C3), i.e., at pH 6.4 and N/P ratio of 75. As shown in Figure 1, the gel electrophoresis assay indicated a partial complexation under these conditions disclosing a binding efficiency between 62.63% and 66.53%. According to data given in Table 2, the observed binding efficiency between PLL and dsDNA ranged from 17.97% to 99.4% depending on the levels of pH and N/P ratio. Overall, the conclusion of the performed optimization experiment was that with the increase of the polyplex N/P ratio, DNA binding performance was also increasing.

On the basis of collected data, a response surface model was developed in terms of two coded variables (*x*_1_ and *x*_2_) by using the multivariate regression method. The fitted model in terms of coded variables is given as:

(1) $$\hat{Y}=66.704-5.351x_{1}+38.581x_{2}+4.234x_{1}^{2}-8.805x_{2}^{2}+1.855x_{1}x_{2}$$

$$\mathrm{subject}\;\mathrm{to}:-1\leq x_{i}\leq +1,\;\forall i=\bar{1,2}$$

The coefficients in (Equation 1) are significant ones according to a Student’s *t*-test [32,33]. The developed model was validated by the analysis of variance (ANOVA) method [30]. Outcomes of ANOVA statistical test are detailed in Table 3.

**Figure 2:**
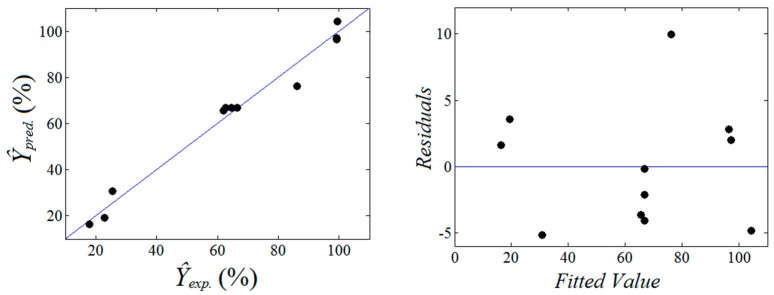
Goodness-of-fit analysis: agreement between experimental observations and calculated predictions (**right**); residual errors versus fitted value (**left**).

The significance of the statistical model is given by the fact that the *p*-value (probability of randomness) is quite low (i.e., *p*-value = 0.000387). In addition, the value of *R*^2^ coefficient (coefficient of determination) shows a good accuracy of the model that is able to explain more than 97% of the data variation. The ability of the model to predict the observed binding efficiency is displayed in a goodness-of-fit graph (Figure 2), highlighting the agreement between predicted and experimental data.

According to Figure 2, the data points are close to the bisector accounting for a good accuracy in predicting the binding efficiency *Ŷ* (%). Both the ANOVA test (Table 3) and the goodness-of-fit plot (Figure 2) suggested a statistical valid model that can be used to explore (by simulation) the designed factorial space describing the complexation process. To develop the data-driven model in terms of actual variables, a substitution technique was applied and the final equation was given as:

(2) $$\hat{Y}=214.522-62.335pH+1.062r+4.234pH^{2}-3.522\times 10^{-3}r^{2}+3.71\times 10^{-2}pH\times r$$

Subject to:25≤r≤125; 5.4 ≤pH≤7.4

**Figure 3:**
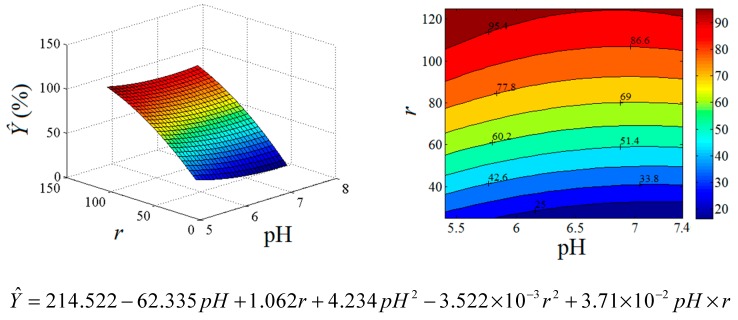
Response surface plot (**left**) and contour-line map (**right**) depicting the effects of pH and N/P ratio (*r*) factors on the binding efficiency *Ŷ* (%).

On the basis of the empirical model (Equation 2), we were able to generate the response surface plot and the contour-lines map (Figure 3) showing the synergetic influence of the input variables (factors) on the binding response (*Ŷ*).

Analyzing the data from Figure 3, we observed that the N/P ratio (*r*) has the most significant effect on the binding efficiency. The second factor (pH) played a more diminished role in the complexation process compared to N/P ratio. Decreasing of the pH value from 7.4 to 5.4 led to a moderate improvement of the binding efficiency, but, overall, the increment of the N/P ratio and the decrease of pH factor resulted in the enhancing of the binding response (*Ŷ*). This fact can be attributed to the complete protonation of the primary amine groups in PLL at lower pH value. Note that the interaction effect between factors (*r* and pH) is a minor one. Predictions provided by the response surface model were in reasonable agreement with experimental data collected from the agarose gel electrophoresis assays.

The process optimization was done by means of the genetic algorithm method implemented in SciLAB (version 5.5.2, Scilab Enterprises, Rungis Complexe, France) for scientific calculations. To this end, Equation 2 was used as objective function for the model-based optimization considering the boundary constraints for the input variables. The found optimal solution converged to *x*_1_ = −1 and *x*_2_ = 1 (coded variables) and, in terms of actual factors, the optimal conditions were pH 5.4 and N/P ratio of 125. The predicted response for optimal conditions was equal to *Ŷ =* 104.21. In turn, the observed binding efficiency (experimental confirmation) was found at its maximal value of *Y* = 99.40%. The difference between the predicted and observed responses is in the limits of the residual error.

The obtained maximum values and the observed tendencies in the above experiments are in accordance to the previously reported investigations on PLL/DNA interactions [17,18,19] and are very important in terms of polyplex formation. The optimum conditions for polyplex preparation, defined by Kang et al [34] as “extracellular medium”, might significantly influence the subsequent in vitro transfection experiments. This extracellular medium used for laboratory cell cultures can be modulated by adding or removing various components and by adjusting the pH to fit specific purposes. The exact and optimized data obtained in vitro could be further used when applying for in vivo experiments where extracellular environments are specific, predominantly affected by pathological differences [35,36]. It is difficult to predict weather the optimum conditions for polyplex formation will greatly influence the transfection results due to the fact that the pH environment affects characteristics of polymers, polyplexes, and cells [34]. We anticipate that understanding the effects of pH values and N/P ratio optimization on polyplex preparation may stimulate new strategies for determining effective and safe polymeric gene carriers.

### 2.2. Molecular Dynamics Simulation of dsDNA/PLL Polyplex Formation

To shed light on dsDNA/PLL molecular interactions at the atomistic level, we performed molecular dynamics (MD) simulations [33] by considering the explicit solvent environment. For this purpose, the modeled dsDNA mimicked the same nucleotide sequence as the one that we used in the experiments.

From the beginning, we should mention the following aspects adopted for the simulation: according to partial PLL amino group protonation in physiological environment due to the neighboring group effect [37] and our experimental observations given in Table 2, the binding efficiency was greater to some extent at pH 5.4 than at pH 7.4, especially for N/P ratio equal to 75 or lower. This fact suggested that protonation degrees of PLL at pH 5.4 and 7.4 might differ in the statistical sense. Because the isoelectric point of PLL is around 9.0 [38], at pH 7.4, this macromolecule obviously carries a net positive charge. Therefore, at pH 7.4, we assumed for modeling purposes, a protonation degree of PLL equal to 50% to explore by simulation the low extent of protonation in the statistical mean. In turn, for pH 5.4, we adopted the full protonation degree of PLL (i.e., 100%). Hence, the half protonation (50%) of PLL (at pH 7.4) is more or less a modeling artifact adopted only for in silico analysis in order to survey by simulation the extreme limit of polyplex formation.

**Figure 4:**
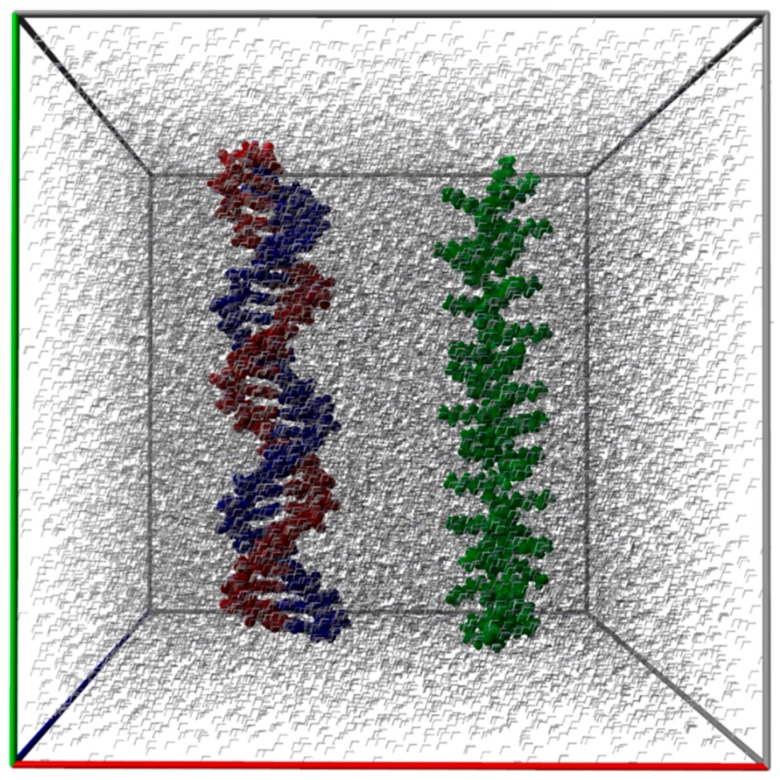
Rendering of initial equilibrated structures of macromolecules dsDNA and PLL in a simulation box with explicit water molecules (solvent), at t = 0 ns.

Figure 4 depicts the initial snapshot (t = 0 ns) of the modeled system showing in an explicit fashion all molecules and atoms, i.e., dsDNA and PLL surrounded by water molecules. The macromolecules (dsDNA and PLL) were separated at the start point by a distance of 40 Å between their centers of geometry (COG distance).

**Figure 5:**
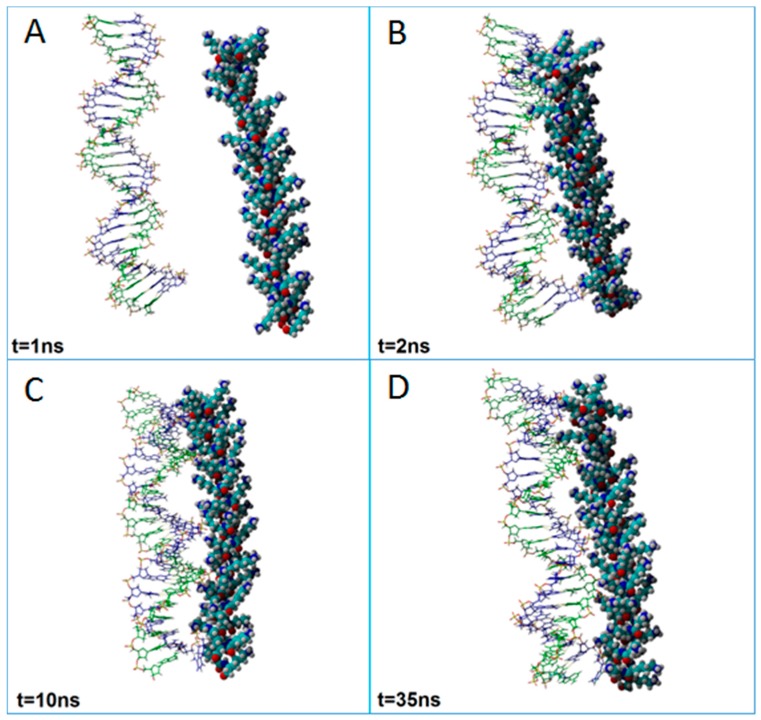
Snapshots from the simulation showing the formation of the polyplex between dsDNA and PLL at a pH value of 5.4 at different simulation times: (**A**) t = 1 ns; (**B**) t = 2 ns; (**C**) t = 10 ns; (**D**) t = 35 ns.

**Figure 6:**
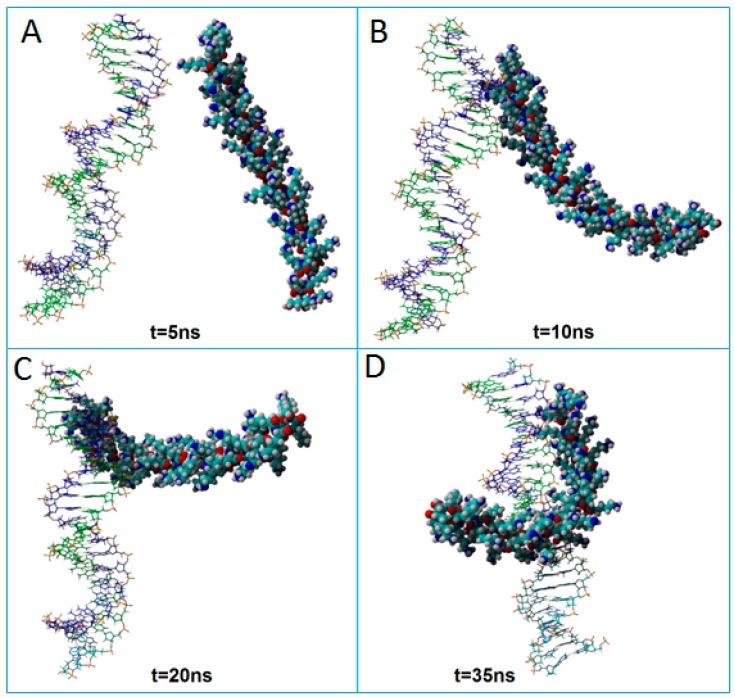
Snapshots showing the interactions between PLL and dsDNA at a pH value of 7.4 with the formation of a polyplex. The time intervals are: (**A**) t = 5 ns; (**B**) t =10 ns; (**C**) t = 20 ns; (**D**) t = 35 ns.

Figure 5 and Figure 6 show typical progress snapshots of molecular dynamics simulations performed at different pH values, i.e., pH 5.4 and pH 7.4, respectively. According to MD simulation results, the pH factor had a central role on the complexation rate (tempo) between dsDNA and PLL. For instance, at pH 5.4, the complexation process was almost complete for t = 2 ns (Figure 5B). In turn, at pH 7.4, the polyplex formation was incomplete even after 20 ns (Figure 6C). This difference is attributed to the adopted degree of protonation of PLL at different pH values. Because PLL was fully protonated at pH 5.4, the molecule showed a high positive charge uniformly distributed across the entire molecule. As a consequence, the entire PLL molecule was aligned near the dsDNA in a parallel fashion (Figure 5B).

Once the first contacts between oligomers emerged at pH 5.4, macromolecules remained in the proximity for the entire period of the simulation with a minimal variance of COG distance (Figure 5). By contrast, at pH 7.4, only a part of PLL (top side) has interacted with dsDNA (Figure 6). This can be attributed to the fact that, at pH 7.4, only 50% of the amine groups were considered protonated in our simulation. Thus, simulation results revealed that, even if a lower protonation degree (50%) of PLL was considered, the dsDNA/PLL polyplex was still formed, but more time was required for its stabilization compared to a full (100%) protonation case.

**Figure 7:**
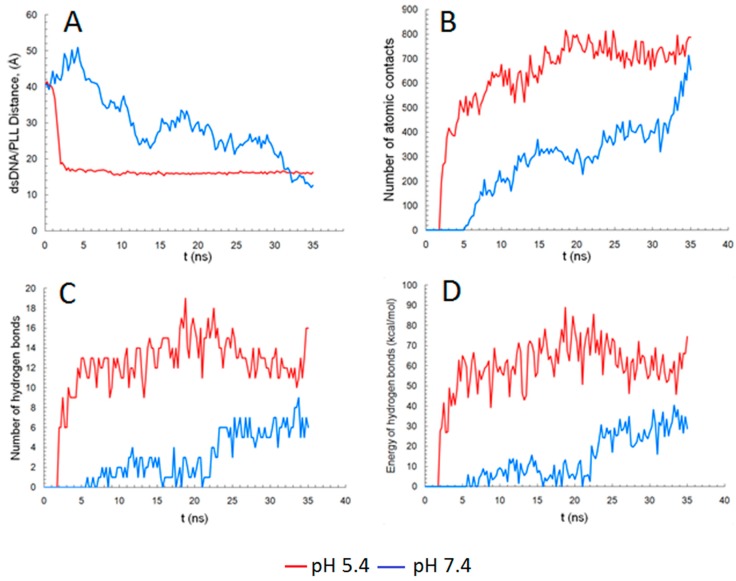
Plots, for dsDNA and PLL, as a function of time at different pH values (red line—pH 5.4; blue line—pH 7.4) of (**A**) the distance between the centers of geometry and (**B**) the number of intermolecular contacts with a cutoff radius of 4 Å, (**C**) number of total hydrogen bonds and (**D**) total energy of hydrogen bonds formed.

Figure 7 displays the history (i.e., evolution in time) of the molecular interaction descriptors between dsDNA and PLL that were recorded in the course of MD simulations. Hence, Figure 7A shows the evolution of the COG distance between the macromolecules at both investigated pH values. As can be seen from Figure 7A, the distance decreased very fast at pH 5.4 (from 40 Å at t = 0 ns to 18 at t = 2 ns), and then stabilized at a value of ~16 Å after 5 ns. In the case of pH 7.4, the polyplex formation followed a different pathway due to the fact that only the top part of the PLL molecule interacted closely with the dsDNA. This led to an initial increase of the COG distance between the macromolecules (at 3.5 ns the distance was about 50 Å, compared to the 40 Å fixed at the start). After 4.5 ns, the distance began to fluctuate with an overall slow descending trend, attaining a value of 16 Å after 32 ns. Figure 7B indicates the number of interatomic contacts that emerged between dsDNA and PLL macromolecules for both of the cases (pH 5.4 and 7.4). In case of pH of 5.4, the first contact occurred just before the 2 ns mark and then the number of contacts increased rapidly to more than 500 at 5 ns. After that, the number of contacts fluctuated as the macromolecules repositioned themselves. However, the general trend was an upward one until 18.5 ns, when a peak was reached (816 contacts). From 18.5 to 35 ns, the number of contacts fluctuated into the interval ranging from 650 to 800. The observed fast closing of the gap between the PLL molecules and the dsDNA is similar to the data reported by Ziebarth et al [17], in which the PLL was considered fully protonated.

Due to the fact that the simulated complexation rate was decelerated at pH 7.4, the first contact between the molecules was observed only at 5 ns. For this case (pH 7.4), the increase of intermolecular contacts was much slower. More precisely, at 18.5 ns, the number of contacts was about 285 and a maximum of 700 contacts was attained only at the final stage, i.e., at t = 35 ns.

According to simulation outcomes, the stability of the polyplex was also influenced by the number of hydrogen bonds that formed between dsDNA and PLL macromolecules. Figure 7C–D highlight the number of hydrogen bonds and their total energy against the simulation time. As shown in Figure 7C, the first hydrogen bonds formed rapidly and in a greater number at pH 5.4 compared to pH 7.4. This may be explained by the different protonation degree of PLL’s nitrogen atoms considered for simulations. The number of hydrogen bonds (Figure 7C) and total energies of H-bonds (Figure 7D) unveiled similar ascending trends as time elapsed. Overall, the difference between the two cases considered might be explained by the fact that, at pH 7.4, the hydrogen bonds, formed between dsDNA and PLL, were less in number and weaker, which led to a totally different conformation of the complex compared to the case of pH 5.4.

Important descriptors used for the characterization of a macromolecule deal with the radius of gyration (*R_g_*) and the root-mean-square-deviation (RMSD) of atomic positions. Such descriptors detail changes that appear in the conformation of a macromolecule or biomolecule providing clues related to its behavior and function [39]. The *R_g_* measures the root-mean-square distance of chain segments from their center of the mass. Thus, *R_g_* is a meaningful macromolecular descriptor that gives a sense of the size of the oligomer/(bio)polymer coil. In turn, the second descriptor (RMSD) compares the current conformation of a simulated macromolecule with the conformation of a target structure. In this study, we considered as the targeted structure the initial equilibrated geometry of the macromolecule at time zero (t = 0 ns). Figure S4 reports the variation of conformational descriptors (*R_g_* and RMSD) during the simulation progress. As shown in Figure S4A,B, the *R_g_* value dropped immediately in both pH conditions and for each macromolecule (i.e., from 26 Å to 24 Å for dsDNA, and from 24 Å to 21 Å for PLL). After the initial drop, *R_g_* fluctuated around a steady value for each macromolecule. Hence, both macromolecules (PLL and dsDNA) were in a slightly compacted states during simulation compared to their initial equilibrated geometries. For dsDNA, the values of *R_g_* fluctuated around 24 Å for both pH conditions (Figure S4B). By contrast, the *R_g_* fluctuated depending on pH level in the case of PLL. More detailed, it has oscillated around 21 Å (at pH 5.4). In turn, at pH 7.4, the radius of gyration associated with PLL dropped to a value of 18 Å (after 20 ns) and fluctuated further near this value (see Figure S4A).

Figure S4C,D highlights the history of RMSD values as the simulation time elapsed. According to Figure S4C, the RMSD values for PLL varied into the limits of 5–7 Å (at pH 5.4) and 5–13 Å (at pH 7.4). As for dsDNA (Figure S4D), the RMSD values varied between 4 and 6 Å (at pH 5.4) and 5–8 Å (at pH 7.4).

It is well known that if the RMSD value is greater than 3 Å, then the molecular conformation is different from the targeted structure. In our case, it is clear that both molecules suffered conformational changes during the complexation process. It should be pointed out that the conformation of PLL macromolecule suffered the most when only 50% of the amine nitrogen atoms were considered protonated. As a consequence, PLL macromolecule was twisted and bended at a greater extent in order to interact with the phosphate groups from dsDNA.

By combining the information obtained from *R_g_* and RMSD plots, one may observe that both dsDNA and PLL are flexible macromolecules, changing their conformations during the complexation process. However, dsDNA still maintained its B-form at the end of the simulation. These outcomes fall in line with the results reported by Ouyang et al. [40], with the difference that various protonated states of the PLL were correlated with the changing in pH.

## 3. Materials and Methods

### 3.1. Materials

Poly(l-Lysine) (PLL) in 0.1% *w*/*v* solution with an average molecular weight 150–300 kDa and used without additional dilution, ethidium bromide, tris (hydroxymethyl)aminomethane, Ethylenediaminetetraacetic acid (EDTA), glacial acetic acid and sucrose were purchased from Sigma-Aldrich (Munich, Germany). Agarose for gel electrophoresis was provided by AppliChem GmbH (Darmstadt, Germany). HPLC purified DNA sequences were purchased from Metabion AG (Planegg/Steinkirchen, Germany), diluted to the concentration of 100 μM and used as a stock solution. The sense strand was 5′-CAAGCCCTTAACGAACTTCAACGTA-3′ and the antisense strand was 5′-TACGTTGAAGTTCGTTAAGGGCTTG-3′.

### 3.2. Polyplex Preparation and Agarose Gel Electrophoresis Assay

*dsDNA stock solution* was prepared by annealing sense and antisense DNA strands in Tris-acetate-EDTA (TAE) buffer at the correspondingly adjusted pH values i.e., 50 μL DNA sense strand, 50 μL DNA antisense strand, 37.5 μL 10×TAE (40 mM Tris, 2 mM acetic acid and 1 mM EDTA) and 18.8 μL NaCl 1 M. The experimental pH values of solutions were chosen as pH 5.4, 6.4 and 7.4.

*Polyplex preparation*: for the calculation of N/P ratio, it was considered that 1 µg of dsDNA contains 3 nmol of phosphate [41], and nitrogen content was determined from the amount of added PLL, considering that MW of a polymeric unit was 128 g·mol^−1^. Thus, for the preparation of polyplexes, the following procedure was performed: 24.6 µL of DNA stock solution was mixed with solution of PLL 0.1% *w*/*v* solution (3 µL was used for N/P ratio of 25; 25, 9 µL for N/P ratio of 75; and 15 µL for N/P ratio of 125), according to a required N/P ratio (Table 1), followed by the addition of 22.5 µL sucrose solution (25% in water). The final volume of the reaction mixture was adjusted to 60 µL with an appropriate amount of water. Samples were incubated for 1 h at 25 °C prior to loading into the gel.

From a prepared polyplex solution, four samples of 15 µL each were loaded onto 1% agarose gel and run at 90 mV (current 700 mA) for 60 min at room temperature in 1×TAE buffer. Subsequently, the gel was stained with ethidium bromide for 15 min at room temperature and then photographed using DNR Bio-imaging system (version 7.0.16, DNR Bio Imaging Systems Ltd, Jerusalem, Israel).

### 3.3. Quantification Methods

In this study, the quantification of the binding affinity between PLL and DNA was performed by analysing the images obtained with the Gel Quant Express software (version 7.0.16, DNR Bio Imaging Systems Ltd) (Figure 1). Typically, a gel electrophoresis experiment at a certain pH value contained dsDNA as a reference whose intensity was quantified by the software as 100%, and several (three or four) parallel samples of a given D/P ratio. The binding efficiency *Y* (%) was calculated by using the following equation [20]:

(3) $$Y=100-I_{bs}$$

where: 100 stand for the intensity of the migrated spot of dsDNA (the reference lane); and *I_bs_* (%) is the average intensity (%) of unbounded dsDNA in the lanes, where samples with certain N/P ratios have been loaded. The exact value of the binding efficiency for each N/P ratio was calculated by averaging the results of 6 different loading samples.

### 3.4. Response Surface Methodology

Response surface methodology (RSM) is a collection of tools, both mathematical and statistical, which is used for data-driven modeling and optimization of experimental processes [42]. This method combines multivariate regression modeling with the design of experiments (DoE) in an efficient way, in order to optimize the input variables (conditions), so they return the best output variables (results) [27,28,29,30,31]. The DoE helps with reducing the number of experimental runs needed to find the optimum conditions, by making possible the simultaneous changing of different variables.

According to RSM, the data-driven model can be constructed based on DoE (collected data) and by using the multivariate regression technique. Generally, the developed model represents a polynomial equation useful to approximate the process performance and it can be expressed as follows [30]:

(4) $$\hat{Y}=b_{0}+\sum_{i=1}^{n}b_{i}x_{i}+\sum_{i=1}^{n}b_{ii}x_{i}^{2}+\sum_{i<j}^{n}b_{ij}x_{i}x_{j}$$

where *Ŷ* denotes the predicted response (i.e., process performance); *x_i_*—coded levels of the input variables; *b_0_*, *b_i_*, *b_ii_*, *b_ij_*—regression coefficients (offset term, main, quadratic and interaction effects). The least square estimations of the coefficients *b* = {*b_0_*, *b_i_*, *b_ii_*, *b_ij_*}^T^ are computed by means of the multivariate regression method and can be written as follows [29,30]:

(5) $$b={\left(X^{T}X\right)}^{-1}X^{T}Y$$

where *b* is a column vector of regression coefficients, *X* is the design matrix of the coded levels of input variables, *X^T^* is the transposed matrix of *X* and Y is a column vector comprising values of the observed response.

### 3.5. Molecular Dynamics Simulations

Molecular dynamics (MD) is a simulation tool employed to comprehend the dynamic structural behavior, functions and interactions of biological macromolecules [33]. Hence, MD provides theoretical information (at the molecular level) regarding the individual motion of atoms in macromolecules versus the simulation time. In the following, the simulation protocol is detailed. The B-form of dsDNA was built for computation purpose by using the YASARA-Structure program (version 14.12.2, YASARA-Biosciences GmbH, Vienna, Austria) (yasara.org). It was constituted from a sense strand 5′-CAAGCCCTTAACGAACTTCAACGTA-3′ and an anti-sense strand 5′-TACGTTGAAGTTCGTTAAGGGCTTG-3′. The final modeled macromolecule (oligomer) was made up of 50 nucleotides with a total charge value of −52 in the fully deprotonated state. The molecular weight was 15.43 kDa and the modeled dsDNA involved terminal phosphate groups at 5′-end position with a negative charge value of −2 (at O1P and O3P). The simulated PLL was built from 1056 atoms summing up a molecular weight of 6.2 kDa and a total charge of +49 in the fully protonated state. Molecular dynamic simulations were performed by means of a YASARA-Structure software package version 14.12.2 (YASARA-Biosciences GmbH, Vienna, Austria) [43] that comprised the “AutoSMILES” algorithm for automatically parameterization of the unknown molecular structures. Hence, this algorithm was used to generate the force field parameters for the molecular dynamic simulations.

According to the adopted simulation protocol, the investigated macromolecules (dsDNA and PLL) were solvated in 32,373 TIP3P water molecules. The applied cell (box) was rectangular in shape with dimensions of 100 Å * 100 Å * 100 Å, containing a total of 99,201 atoms. The simulation box was set to periodic boundary condition. The first step of simulation dealt with the cell neutralization, followed by the addition of monovalent counterions (Na^+^ Cl^−^) attaining a mass faction of 0.9%. Next, the system was subjected to energy minimization by means of the steepest descent algorithm, simulated annealing optimization and a quick equilibration via the short molecular dynamics computation (2 ps). The resulted conformations were used as the starting point for the MD simulation production run. Note that PLL was considered fully protonated (100%) at pH 5.4 and half protonated (50%) at pH 7.4. Molecular dynamics simulations were performed using the self-parameterizing knowledge-based Yasara force field. The pressure control over the modeled system was enabled by setting the probe mode for the solvent. In other words, the water density was set to 0.997 g·cm^–3^ in order to simulate a constant pressure of *P* = 1 bar at the temperature equal to *T* = 298 K. Newton's equations of motion (SUVAT) were integrated at a time step of 1 fs. Electrostatic interactions were modeled using the particle mesh Ewald (PME) method. All non-covalent interactions between macromolecules (i.e., van der Waals and electrostatic) were computed for a cut-off distance set to 12 Å. Finally, 35 ns long molecular dynamics simulations were carried out twice: (1) for pH 5.4 and (2) for pH 7.4. The trajectories from MD simulations were saved as snapshots every 10,000 steps. The outcomes visualization and trajectory analysis were evaluated by the YASARA program.

Limitations of the MD simulation are generally related to the constrained simulation time scale and the force field accuracy. MD simulations require short time steps (typically from 1 to 5 femtoseconds) for numerical integration of the equations of motion. Therefore, millions of sequential time steps are employed to attain a simulation time of 10 nanoseconds, and even more for the microseconds scale. Enabling longer-timescale MD-simulations is an active research domain, comprising algorithmic improvements, parallel computing and specialized hardware. Although molecular mechanics force fields are inherently mathematical approximations, they have improved substantially over the last decades. For instance, Yasara is a current force field with a good accuracy for modeling of macromolecules/biomolecules.

## 4. Conclusions

In summary, a design of experiments was used to investigate the polyplex formation between short double-stranded oligonucleotide (25 bp) and poly(l-lysine) (150–300 kDa) cationic polymer under various levels of key factors: N/P ratio of PLL/dsDNA polyplex and pH. The degree of complexation between dsDNA and PLL was quantified by processing images obtained from the gel electrophoresis assays using the Gel Quant express software. A multivariate regression model was constructed using design of experiments (collected data) and the responsive surface methodology. The developed model was validated using an ANOVA statistical test. The data-driven model enabled the establishment of the functional relationship between the key factors and binding efficiency (response). The optimal conditions for attaining the maximal binding efficiency (99.4%) were found to be pH 5.4 and with an N/P ratio of 125.

To unveil the behavior of macromolecules and the mechanism of polyplex formation, we performed a set of MD simulations for two cases related to different protonation degrees of PLL. Computational MD outcomes revealed that the binding rate between macromolecules differed with the variation of PLL protonation. Thus, at pH 5.4 (i.e., 100% PLL protonation), the distance between macromolecules decreased from 40 Å to around 18 Å in just few nanoseconds. In turn, at pH 7.4 (i.e., 50% PLL protonation), the same distance was achieved only after 31 ns.

In addition, simulation data indicated that hydrogen bonds were formed predominantly between the backbone oxygen atoms of dsDNA and hydrogen atoms of the amine groups from PLL. In addition, the strength of the bonds was stronger at a pH of 5.4, compared to 7.4. Considering that polyplex formation and stability is strongly related to hydrogen bonds formation, determining the number and strength of hydrogen bonds through MD can offer insights on the theoretical efficiency of the polyplex. On the basis of *R_g_* and *RMSD* values, the flexibility degrees of both macromolecules were ascertained. Computational results revealed that the PLL macromolecule was the most flexible one, which can be bent and twisted to a greater extent at physiological pH value, while DNA conformation was minimally perturbed within the polyplex. Based on the results presented above, we can conclude that, in order to increase the binding efficiency of PLL and increase the loading of DNA in a PLL-based polyplex, decreasing the pH is an effective method.

## Figures and Tables

**Table 1:** Design variables and their coded and real values used for determination of the dsDNA/PLL complexation process.

Design Variables (Factors)	Coded Variables	Real Values of Coded Levels		
		−1	0	+1
Initial pH of solution	*x*_1_	5.4	6.4	7.4
N/P ratio, *r*	*x*_2_	25	75	125

**Table 2:** Faced-centered experimental design used for the investigation of the condensation process between dsDNA and PLL and the experimental result (binding efficiency) determined for each run.

Run Nr	Type ^a^	Design Variables				Binding Efficiency (Experimental)
		pH Solution		N/P Ratio		
		pH (Actual)	*x*_1_ (Coded)	*r* (Actual)	*x*_2_ (Coded)	*Y*(%)
1	F1	5.4	−1	25	−1	25.58
2	F2	7.4	+1	25	−1	17.97
3	F3	5.4	−1	125	+1	99.40
4	F4	7.4	+1	125	+1	99.21
5	A1	5.4	−1	75	0	86.28
6	A2	7.4	+1	75	0	61.97
7	A3	6.4	0	25	−1	22.87
8	A4	6.4	0	125	+1	99.30
9	C1	6.4	0	75	0	64.58
10	C2	6.4	0	75	0	62.63
11	C3	6.4	0	75	0	66.53

F: Factorial value; A: Axial value; C: Central value.

**Table 3:** Analysis of variance (ANOVA) for the significance of the multivariate regression model.

Source	DF ^(a)^	SS ^(b)^	MS ^(c)^	*F*-Value ^(d)^	*p*-Value ^(e)^	*R^2^* ^(f)^	*R_adj_^2^* ^(g)^
Model	5	9.323 × 10^3^	1.865 × 10^3^	44.148	0.000387	0.978	0.956
Residual	5	211.174	42.235				
Total	10	9.534 × 10^3^					

^(a)^ Degree of freedom; ^(b)^ Sum of squares; ^(c)^ mean square; ^(d)^ ratio between mean square; ^(e)^ probability of randomness; ^(f)^ coefficient of determination; ^(g)^ adjusted coefficient of determination.

## Supplementary Materials

The following are available online at www.mdpi.com/1422-0067/18/6/1291/s1.
