# Supplementary material for: Optimization of Polyplex Formation between DNA Oligonucleotide and Poly(l-Lysine): Experimental Study and Modeling Approach
Source: Int J Mol Sci. 2017 Jun 17;18(6):1291. doi: 10.3390/ijms18061291 (PMC5486112; doi:10.3390/ijms18061291)
Supplement: Supplementary file 1 [file ijms-18-01291-s001.pdf]

Optimization of Polyplex Formation between DNA Oligonucleotide and Poly(l-Lysine): Experimental Study and Modeling Approach

Tudor Vasiliu, Corneliu Cojocaru, Alexandru Rotaru, Gabriela Pricope, Mariana Pinteala and Lilia Clima

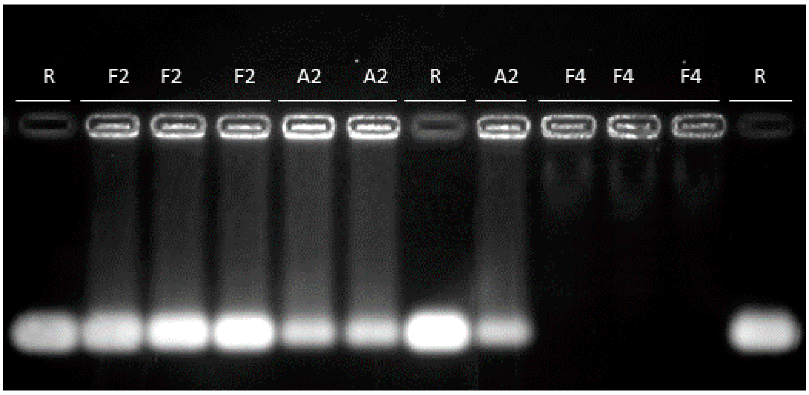

Figure S1. Electrophoresis assay at pH 7.4 where R represents ds DNA, lanes F2, A2 and F4 correspond to PLL/dsDNA complex formation at different N/P ratios according to Table 2.

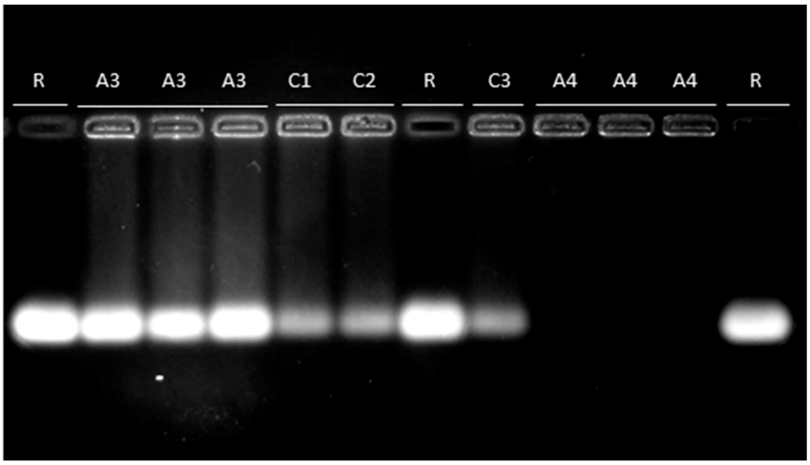

Figure S2. Electrophoresis assay at pH 6.4 where R represents ds DNA, lanes F3, A4 and C1-3 correspond to PLL/dsDNA complex formation at different N/P ratios according to Table 2.

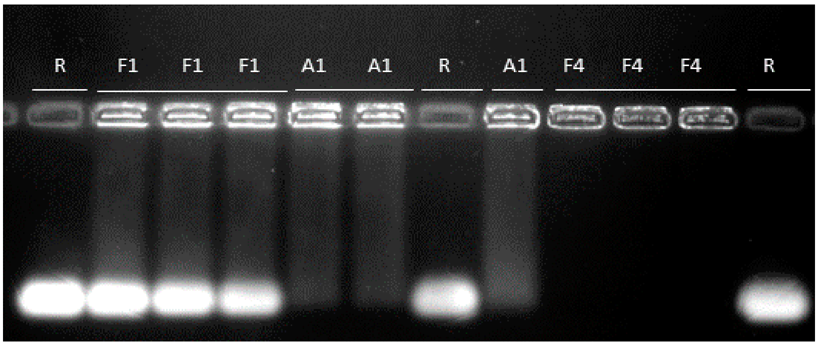

Figure S3. Electrophoresis assay at pH 5.4 where R represents ds DNA , lanes F1, F4 and A1 correspond PLL/dsDNA complex formation at different N/P ratios according to Table 2.

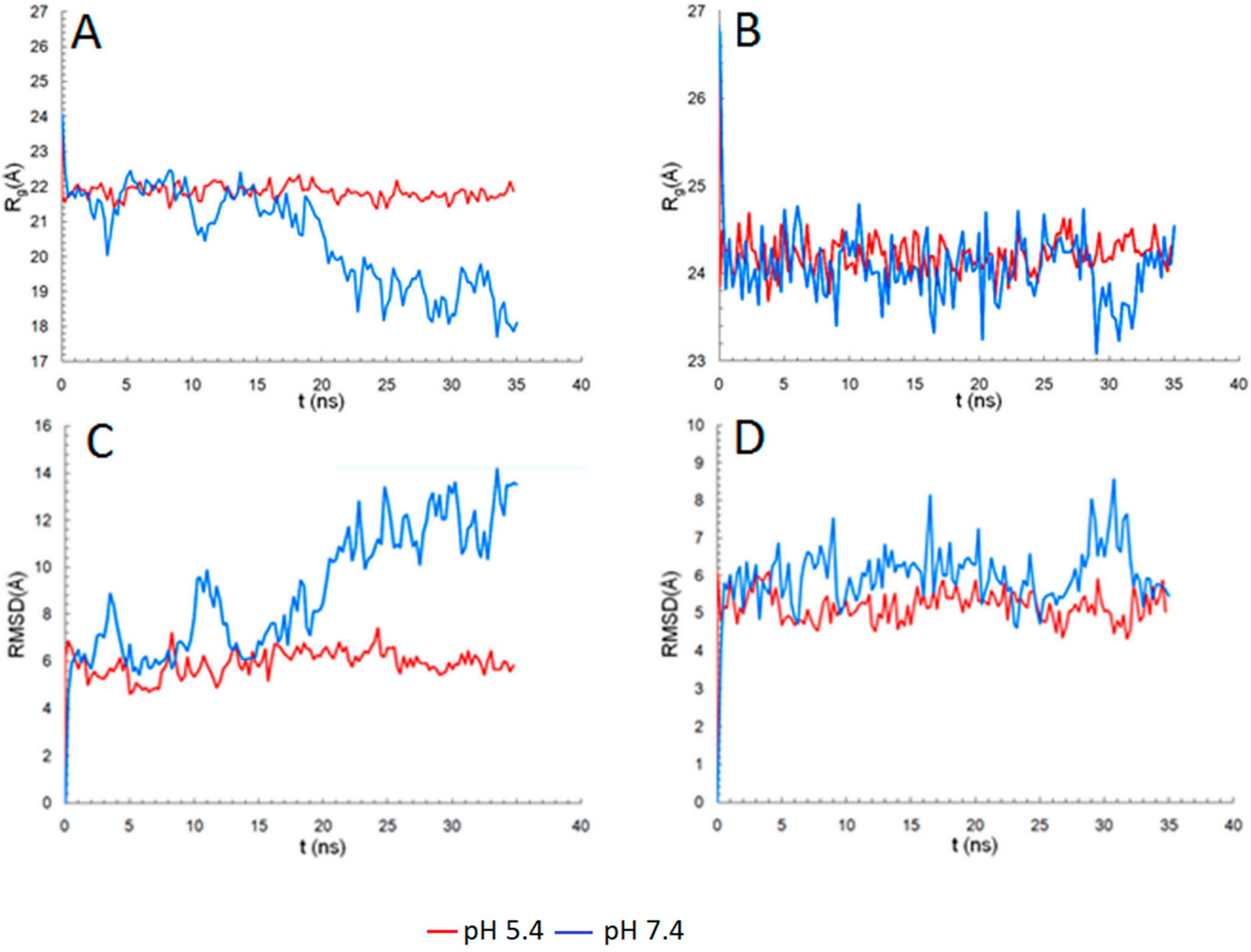

Figure S4. Plots of radius of gyration versus time for macromolecules at different pH: A) PLL and B) DNA, and plots of root-mean-square deviation versus time for C) PLL and D) DNA at pH values of 5.4 and 7.4.
